# The role of habitual physical activity on arterial stiffness in elderly Individuals: a systematic review and meta-analysis

**DOI:** 10.20463/jenb.2017.0041

**Published:** 2017-12-31

**Authors:** Wonil Park, Hun-Young Park, Kiwon Lim, Jonghoon Park

**Affiliations:** 1. Exercise Nutrition and Biochemistry Laboratory, Department of Physical Education, Korea University, Seoul Republic of Korea; 2. Physical Activity and Performance Institute (PAPI), Konkuk University, Seoul Republic of Korea; 3. Department of Physical Education, Konkuk University Republic of Korea

**Keywords:** arterial stiffness, habitual physical activity, elderly individual, meta-analysis, heterogeneity, and random effect model

## Abstract

**[Purpose]:**

Physical inactivity behavior at middle age or older is a major risk factor for cardiovascular disease. However, the effects of levels of habitual physical activity on arterial stiffness in elderly population remain unclear currently. Therefore, the purpose of this study was to demonstrate whether the effects of habitual physical activity could attenuate arterial stiffness in elderly individuals via a meta-analysis.

**[Methods]:**

We searched the Medline and Embase databases from January 1997 through November 2017, using the medical subject headings “older population”, “physical activity” (e.g., walking, cycling, climbing, and any participation in sports), “arterial stiffness”, “pulse wave velocity”, and “cardiovascular health” published in English. Six articles (2,932 participants) were included in this meta-analysis. We investigated the effects of habitual physical activity on arterial stiffness, which was measured by the pulse wave velocity.

**[Results]:**

Results confirmed heterogeneity (Q-value = 160.691, *p* = 0.000, I^2^ = 96.888) between individual studies. The effect size was calculated using random effect model. It has shown that physically active individuals have significantly lower arterial stiffness than their sedentary peers do (standardized mean difference: -1.017 ± 0.340, 95% confidence interval: -1.684 ~ -0.350, p = 0.003).

**[Conclusion]:**

Findings of our systematic review and meta-analysis indicate that habitual physical activity can significantly ameliorate arterial stiffness in the elderly population.

## INTRODUCTION

Cardiovascular disease (CVD) has been the leading cause of mortality and morbidity worldwide for the past years ^1^. In addition, CVD is the cause of more than 800,000 deaths annually and accounts for 40% of all-cause mortality in developed countries ^2^. The majority of CVD is caused by risk factors that could be controlled, or modified, such as hypertension, cholesterol, overweight/ obesity, tobacco use, physical inactivity, and diabetes ^3^. Particularly, the lack of physical activity is ranked fourth, among the risk factors, and causes an estimated 3.2 million deaths worldwide ^4^. Recent studies support the idea that the level of physical activity is inversely related to coronary heart disease (CHD) ^4^ and CVD morbidity or mortality ^5^. Although limited data exists for older adults, physical activity has demonstrated to be beneficial in cardiovascular health. 

Physical activity is defined as any movement produced by skeletal muscles requiring energy expenditure in the body (e.g., walking, cycling, or participating in sports). Habitual physical activity has a favorable effect on health that can reduce the risk of cardiovascular diseases, diabetes, some cancers, and depression ^6^. Sedentary behavior at middle age or older is a major risk factor. Reports show that in physically active persons, moderate amounts of physical activity results in a 20% lower risk, while higher amounts result in an approximately 30% lower risk. Older adults should adhere to the physical activity guidelines advisory committee report 2008, which recommends at least 150 minutes per week of moderate (75 minutes vigorous) physical activity ^7^^-^^9^. However, what is lacking is a meta-analysis study on whether habitual physical activity has a positive effect on cardiovascular health in older adults. Worth noting though, is that, when older adults do exercise, they should maintain or improve balance if they are at risk of falling and they should determine their level of physical activity relative to their physical fitness. Also, older adults with chronic conditions should perform their regular physical activity safely ^10^. 

Arterial stiffness is increased in advancing age, and has been recognized as an independent risk factor for cardiovascular disease. Pulse wave velocity (PWV) is recognized as the gold standard measurement of arterial stiffness, thus a higher PWV is known as a significant predictor of cardiovascular morbidity and mortality in older subjects ^11^. A number of investigators have reported that regular exercise delays the development of age-related increases in arterial stiffness ^4^^,^^12^. Men who had higher physical conditioning status have lower arterial stiffness indices, both carotid AI (augmentation index) and aortic PWV, than their less active agematched peers ^12^. However, the effect of habitual physical activity (such as steps per day, or time spent on moderate to vigorous activity per day) on arterial stiffness among older populations are very limited. Although the trend of age-associated increases in arterial stiffness is well established ^13^^-^^16^, it remains unclear whether habitual physical activity mitigates arterial stiffening in older adults. Therefore, the primary aim of this systematic review with meta-analysis is to pool results from emerging evidence of clinical studies to clarify relationship between physical activity and arterial stiffness in older adults. 

## METHODS

### Reference search and data extraction

The systematic review was conducted according to the Cochrane guidelines and is reported according to PRISMA guidelines ^17^^,^^18^. We primarily searched MEDLINE and EMBASE databases from January 1997 through November 2017, using the medical subject headings “older population”, “physical activity” (e.g., walking, cycling, climbing, and any participation in sports), “arterial stiffness”, “pulse wave velocity”, and “cardiovascular health”, published in English. We excluded as follows: 1) prescribed structured exercise intervention and 2) patients with any chronic disease. All titles and abstracts of identified publications were screened independently by two reviewers. The full texts of relevant publications were assessed by the same reviewers. Of the 1,286 initial search shows, 1,273 articles were excluded for common reasons including inappropriate measure of arterial stiffness, non-specific age, and patients with clinical history. We reviewed all relevant articles and identified 13 published studies of habitual physical activity and arterial stiffness, 6 of which met our inclusion criteria. Inclusion criteria were, English language reports of any cross-sectional or intervention study. 

### Statistical analysis

All statistical analyses were performed with Excel (Microsoft, USA) and CMA version 3.0 (Biostat, USA). Cohen’s d was used to calculate the effect size in individual studies, i.e., to calculate the standardized mean difference ^19^. Cohen’s d divides the mean difference by the merged standard deviation of the two groups and categorizes the value of the effect size; it has a small effect when it is 0.2 or more, a moderate effect when it is 0.5 or more, and a large effect when the d value is 0.8 or more. However, since Cohen’s d tends to overestimate the effect size when the sample size is small, Hedges’g is needed to correct this, and when the sample size is a mixture of large studies and small studies, it is necessary to convert Cohen’s d to Hedges’ g ^20^. This meta-analysis calculated the effect size of studies that converted from Cohen’s d to Hedges’ g with correction factor. The homogeneity assessment of effect size in individual studies were evaluated by Q-value (*p* > 0.10) and Higgins' I^2^ (< 50%) statistics. Under the fixed-effect model we calculated the weighted effect size (weighted mean difference: WMD) if the test of homogeneity was statistically significant, and vice versa, we employed the random effect model. A significance level of a < 0.05 was used to determine statistical difference for mean of effect size, and the confidence interval (CI) was reflected at a confidence level of 95 %. 

## RESULTS

**Figure 1.**
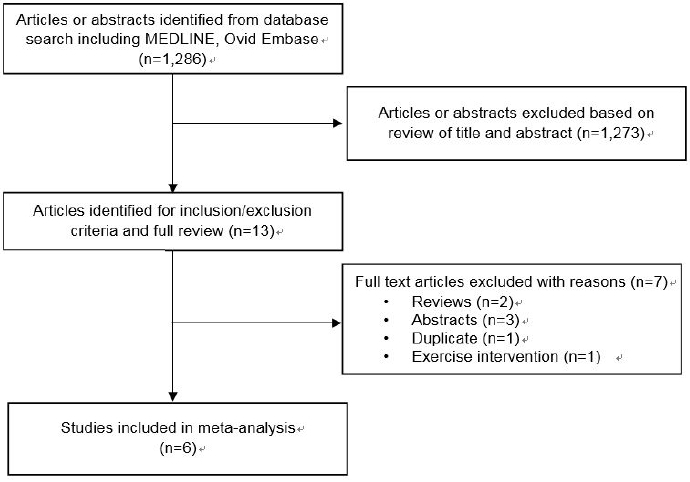
Flowchart of selection of studies for inclusion in this systematic review and meta-analysis.

The flow through the selected studies is summarized in Figure 1. Characteristics of the studies included in the systematic review and meta-analysis are summarized in Table 1. 

**Table 1:** Characteristics of selected studies in the systematic review and meta-analysis.

Author	Sample size (n)	Measurement of PA	Age (years)	BMI (kg/m2)	SBP (mmHg)	DBP (mmHg)
Gonzales et al. (2015) [34]	47	Accelerometer	65.1 3.8	22.4 2.3	115.0 13.6	66.5 7.4
Aoyagi et al. (2010) [35]	198	Accelerometer	+5 4.6	23.0 3.0	136.0 17.0	83.0 17.0
Endes et al. (2016) [36]	1,898	Questionnaire	60.9 10.6	25.4 5.3	-	-
Sampaio et al. (2014) [16]	175	Questionnaire	76.0 7.0	20.7 2.61	-	-
Gando et al. (2010) [37]	109	Accelerometer	63.0 0.3	22.2 0.4	118.0 2.0	70.0 1.0
Crichton et al. (2014) [38]	505	Questionnaire	58.8 12.2	26.2 4.3	116.8 18.4	65.1 8.4

Values are mean S.D or SE. PA; physical activity, BMI; body mass index, SBP; systolic blood pressure, DBP; diastolic blood pressure.

**Figure 2.**
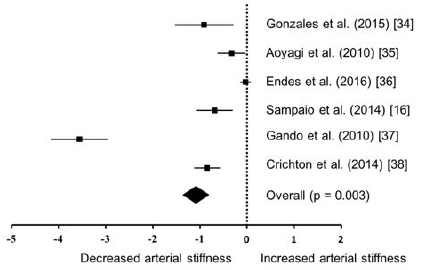
Hedges’ g for standardized mean difference and 95%confidence interval for habitual physical activity and arterial stiffness in meta-analysis.

As shown in Table 2 and Figure 2, we used a PRISMA flow chart to select six studies and then performed a meta-analysis to verify the difference in arterial stiffness between physically active and sedentary elderly individuals. Consequently, heterogeneity (Q-value = 160.691, *p* = 0.000, I^2^ = 96.888) in arterial stiffness was confirmed between individual studies, and the effect size was calculated using random effect model. It has shown that physically active individuals have significantly lower arterial stiffness than their sedentary peers do, according to their standardized mean difference: -1.017 ± 0.340, 95% CI: -1.684 ~ -0.350, p = 0.003). 

**Table 2:** Difference between arterial stiffness in healthy vs sedentary persons.

Author	Standardized mean difference (Hedges’ g)	standard error	variance	Lower limit	Upper limit	Z-value	p-value
Gonzales et al. (2015) [34]	-0.910	0.312	0.097	-1.521	- 0.299	- 2.920	0.003
Aoyagi et al. (2010) [35]	-0.326	0.148	0.022	- 0.616	- 0.037	- 2.210	0.027
Endes et al. (2016) [36]	-0.033	0.054	0.003	- 0.139	0.074	- 0.598	0.550
Sampaio et al. (2014) [16]	-0.688	0.192	0.037	-1.064	- 0.312	- 3.588	0.001
Gando et al. (2010) [37]	-3.559	0.307	0.094	- 4.161	- 2.957	- 11.590	0.001
Crichton et al. (2014) [38]	-0.845	0.136	0.018	-1.111	- 0.579	- 6.231	0.001
Overall	-1.017	0.340	0.116	-1.684	- 0.350	- 2.989	0.003

## DISCUSSION

International hypertension societies recommend longterm physical activity in older adults, as a lifestyle-modification to improve blood pressure control and reduce the risk of cardiovascular disease ^21^. Our pooled data indicates that the arterial stiffness of older people shows an inverse association with habitual physical activity. To our knowledge, this is the first meta-analysis to show the association of arterial stiffness and habitual physical activity in older population without any clinical cardiovascular disease. Although previous studies related with exercise intervention showed similar findings, this study shows that habitual physical activity has favorable effect on arterial stiffness in older population. 

The elderly might experience several adverse health outcomes as a result of vascular aging, such as increased arterial stiffness, which can contribute to the development of cardiovascular and cerebrovascular diseases ^22^. In this context, physical activity may be a cost-effective reduction strategy for those who cannot use or afford medications, or who cannot access traditional therapeutic choices ^23^. Considering the multitude of benefits induced by habitual physical activity, it seems logical that the detrimental effects of aging on vascular function could be antagonized through participation in habitual physical activity ^24^^-^^29^. Besides, habitual physical activity and exercise training both appear to limit age-related increases in arterial stiffness, in addition to improving many other points of human health ^30^. Kozakova et al. (2007) demonstrated the relationship between habitual physical activity as measured by the accelerometer, and arterial stiffness in healthy adults. They found that arterial stiffness was inversely correlated with habitual physical activity, based on the multiple regression models in both males and females ^31^. Sugawara et al. (2006) reported that moderate to vigorous physical activity has favorable effects on arterial stiffness, although physical activity with light intensity had no such effect ^32^. In line with this, age-related increase in central arterial stiffness was not shown in physically active women ^12^. Through habitual physical activity, several underlying mechanisms leading to arterial stiffness via structural (e.g. elastin and collagen content) and functional changes (e.g. fracture and fragmentation of elastic lamellae, and extracellular matrix) are altered ^33^. Therefore, it is thought that habitual physical activity may act to suppress the development of age-dependent arterial stiffness with aging. 

In our meta-analysis, habitual physical activity reduced arterial stiffness as opposed to sedentary lifestyle. Gonzales et al. (2015) investigated whether the level of fatigability during walking contributes to increased arterial stiffness in 45 community-dwelling women and men (60- 78 yrs) ^34^. The change in perceived fatigue was measured after a fast-pace 400-meter walking test and they measured arterial stiffness by carotid-femoral pulse wave velocity (cfPWV). Arterial stiffness was higher (p=.01) for adults with a greater perceived fatigability (10.3 2.1 m/s) than lower fatigability (8.4 2.0 m/s). Another study showed that arterial stiffness was significantly lower in physically active individuals, apparently reaching a minimum in subjects who exceeded counts of about 6,600 steps/day and/or exercise for more than 16 min/day at an intensity >3 METs ^35^. Endes et. al. (2016) investigated whether habitual physical activity is associated with lower arterial stiffness in older adults ^36^. They examined a cross-sectional study of self-reported physical activity intensities with arterial stiffness in elderly Caucasians of the Swiss Cohort Study on Air Pollution and Lung and Heart Disease in Adults. They demonstrated that higher levels of self-reported physical activity are associated with lower arterial stiffness in the elderly, compared with lower levels of physical activity, even after adjustment for several confounding factors. In addition, arterial stiffness is associated with low physical activity levels in Japanese community-dwelling older adults ^15^. They recruited 175 community-dwelling Japanese older adults through questionnaires and arterial stiffness. The results indicated that the older adults with lower physical activity levels had higher arterial stiffness compared with normal subjects. Gando et al. (2010) showed a significant interaction between age and time spent in light physical activity, in determining carotid-femoral pulse wave velocity (p < 0.05) ^37^. In other words, arterial stiffness was lower in the high to light physical activity level than in the low to light physical activity level. These results suggested that longer time spent in light physical activity is associated with deterioration of arterial stiffness. Lastly, the American Heart Association (AHA) health metrics consists of four health behaviors (smoking, body mass index, physical activity, and diet) and three health factors (total cholesterol, blood pressure, and fasting plasma glucose) ^38^, Based on which the cardiovascular health score (CHS) is calculated; the better CHS with physical activity is related to lower arterial stiffness. Taken together, t habitual physical activity is considered a relevant index of arterial stiffness in healthy older population; particularly, the effect of habitual physical activity may be enhanced with higher intensity level of physical activity. 

Previous investigations in this study have been weakened because investigators used a subjective questionnaire to estimate overall physical activity ^39^^,^^40^. Therefore, the subjective interpretation of questions and perception of physical activity may have lead to misclassification of the magnitude of activity ^40^. In addition to the imprecision associated with such measures, it is also difficult to determine the amount of light physical activity, such as housework (i.e., sweeping, mopping, and window washing) and other unstructured activities, by questionnaires ^37^. Further studies are needed to demonstrate the relationship between arterial stiffness and habitual physical activity with objective measurements (e.g. accelerometer and pedometer) of levels of physical activity. There are several other limitations in our systematic review and meta-analysis study. First, the sample size of the included studies may have been relatively small and may not provide enough evidence for generalization. However, this may aid in generalizing our results to the healthy general population with advancing age. Second, our meta-analysis was based on summary data from each of the original studies. Therefore, we could not access individual data, which may have provided more information regarding potential factors. 

## CONCLUSIONS

In conclusion, the results of the systematic review and meta-analysis study indicate that habitual physical activity is an effective index of arterial stiffness in healthy older population. The effect of physical activity may be enhanced with higher intensity level of physical activity. Further studies are needed to investigate the effect of various aspects of physical activities on arterial stiffness among older population. 
